# Supplementary material for: Drug‐like sphingolipid SH‐BC‐893 opposes ceramide‐induced mitochondrial fission and corrects diet‐induced obesity
Source: EMBO Mol Med. 2021 Jul 7;13(8):e13086. doi: 10.15252/emmm.202013086 (PMC8350895; doi:10.15252/emmm.202013086)
Supplement: Supplementary file 1 — Appendix [file EMMM-13-e13086-s003.pdf]

APPENDIX
Table of contents

Figure S1 1
Table of exact P values for figures. 3

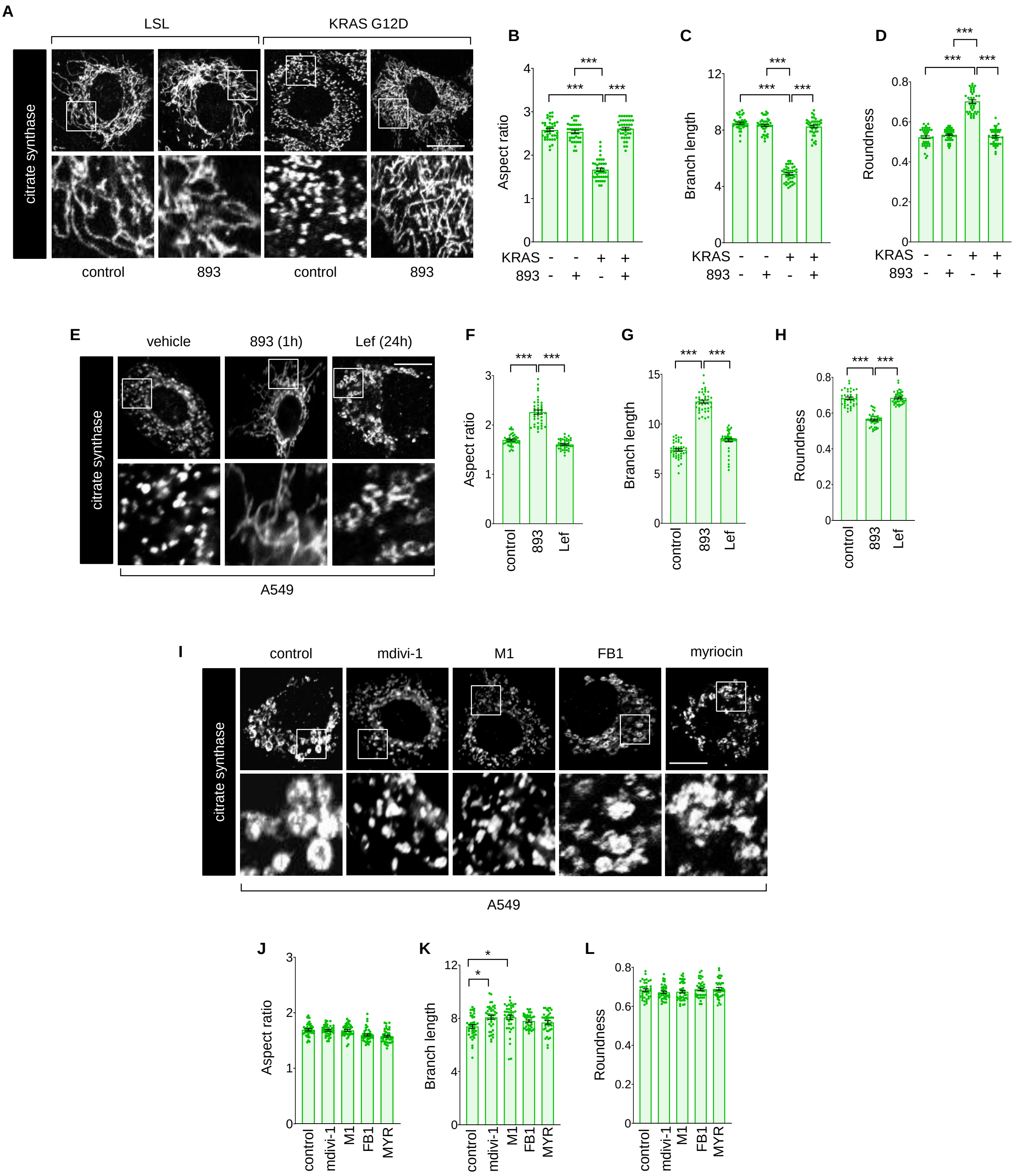
A
LSL
KRAS G12D
citrate synthase
control
893
control
893
B
Aspect ratio
KRAS - - + +
893 - + - +
C
Branch length
KRAS - - + +
893 - + - +
D
Roundness
KRAS - - + +
893 - + - +
E
vehicle
893 (1h)
Lef (24h)
citrate synthase
A549
F
Aspect ratio
control
893
Lef
G
Branch length
control
893
Lef
H
Roundness
control
893
Lef
I
control
mdivi-1
M1
FB1
myriocin
citrate synthase
A549
J
Aspect ratio
control
mdivi-1
M1
FB1
MYR
K
Branch length
control
mdivi-1
M1
FB1
MYR
L
Roundness
control
mdivi-1
M1
FB1
MYR

Figure S1: SH-BC-893 opposes KRAS-induced mitochondrial fission more effectively than other agents. A, Control lox-stop-lox (LSL) or KRASG12D MEFs were treated with SH-BC-893 (5 μM) for 3 h and stained for citrate synthase. **B-D**, Aspect ratio (**B**), branch length (**C**), or roundness (**D**) of mitochondria in the cells in (**A**). **E-H**, A549 cells were treated with vehicle (methanol) or 893 (5 μM) for 1 h or leflunomide (50 μM) for 24 h and mitochondrial morphology quantified as in (**A-D**). **I-L**, A549 cells were treated with vehicle (DMSO), mdivi-1 (50 μM) for 24 h, M1 (5 μM) for 24 h, fumonisin B1 (30 μM) or myriocin (10 μM) for 3 h and and mitochondrial morphology quantified as in (**A-D**). In **B-D**, **F-H**, and **J-L**, 40 cells were evaluated from 2 biological replicates, mean ± SD shown. Using a one-way ANOVA and Tukey's correction, *, $P < 0.05$; ***, $P \leq 0.001$; unmarked, not significant, $P > 0.05$. Scale bars, 20 μm.

EXACT P VALUES FOR ALL FIGURES

	P value	significance
Figure 1B		
BSA vs. PA	<0.0001	****
BSA vs. PA:YM	<0.0001	****
BSA vs. PA:NAV	<0.0001	****
BSA vs. 893:PAL	0.509	ns
PA vs. PA:YM	<0.0001	****
PA vs. PA:NAV	<0.0001	****
PA vs. 893:PAL	<0.0001	****
PA:YM vs. PA:NAV	0.003	**
PA:YM vs. 893:PAL	<0.0001	****
PA:NAV vs. 893:PAL	0.0002	***
Figure 1C		
BSA vs. PA	<0.0001	****
BSA vs. PA:YM	<0.0001	****
BSA vs. PA:NAV	<0.0001	****
BSA vs. 893:PAL	0.0157	*
PA vs. PA:YM	<0.0001	****
PA vs. PA:NAV	<0.0001	****
PA vs. 893:PAL	<0.0001	****
PA:YM vs. PA:NAV	0.9816	ns
PA:YM vs. 893:PAL	<0.0001	****
PA:NAV vs. 893:PAL	<0.0001	****
Figure 1D		
BSA vs. PA	<0.0001	****
BSA vs. PA:YM	<0.0001	****
BSA vs. PA:NAV	<0.0001	****
BSA vs. 893:PAL	0.8167	ns
PA vs. PA:YM	<0.0001	****
PA vs. PA:NAV	<0.0001	****
PA vs. 893:PAL	<0.0001	****
PA:YM vs. PA:NAV	>0.9999	ns
PA:YM vs. 893:PAL	0.0004	***
PA:NAV vs. 893:PAL	0.0004	***
Figure 1F		
BSA vs. PA	<0.0001	****
BSA vs. c16 cer	<0.0001	****
BSA vs. 893:PA 1 h pre-treat	0.0026	**
BSA vs. 893:c16 cer 1 h pre-treat	>0.9999	ns
PA vs. c16 cer	0.5051	ns
PA vs. 893:PA 1 h pre-treat	<0.0001	****
PA vs. 893:c16 cer 1 h pre-treat	<0.0001	****
c16 cer vs. 893:PA 1 h pre-treat	<0.0001	****
c16 cer vs. 893:c16 cer 1 h pre-treat	<0.0001	****

893:PA 1 h pre-treat vs. 893:c16 cer 1 h pre-treat	0.0026	**
Figure 1G		
BSA vs. PA	<0.0001	****
BSA vs. c16 cer	<0.0001	****
BSA vs. 893:PA 1 h pre-treat	0.0433	*
BSA vs. 893:c16 cer 1 h pre-treat	<0.0001	****
PA vs. c16 cer	>0.9999	ns
PA vs. 893:PA 1 h pre-treat	<0.0001	****
PA vs. 893:c16 cer 1 h pre-treat	<0.0001	****
c16 cer vs. 893:PA 1 h pre-treat	<0.0001	****
c16 cer vs. 893:c16 cer 1 h pre-treat	<0.0001	****
893:PA 1 h pre-treat vs. 893:c16 cer 1 h pre-treat	<0.0001	****
Figure 1H		
BSA vs. PA	<0.0001	****
BSA vs. c16 cer	<0.0001	****
BSA vs. 893:PA 1 h pre-treat	0.0073	**
BSA vs. 893:c16 cer 1 h pre-treat	0.8194	ns
PA vs. c16 cer	0.5496	ns
PA vs. 893:PA 1 h pre-treat	<0.0001	****
PA vs. 893:c16 cer 1 h pre-treat	<0.0001	****
c16 cer vs. 893:PA 1 h pre-treat	<0.0001	****
c16 cer vs. 893:c16 cer 1 h pre-treat	<0.0001	****
893:PA 1 h pre-treat vs. 893:c16 cer 1 h pre-treat	0.1424	ns
Figure 1I		
Vehicle	0.0009	***
893	0.0018	**
Myriocin	0.1443	ns
Fumonisin B1	0.4298	ns
Figure 1K		
BSA vs. PA	<0.0001	****
BSA vs. 893	0.9989	ns
BSA vs. 893PA	0.5568	ns
PA vs. 893	<0.0001	****
PA vs. 893PA	<0.0001	****
893 vs. 893PA	0.8708	ns
Figure 1M		
BSA vs. BSA+893	0.3989	ns
BSA vs. PA	0.7932	ns
BSA vs. PA+893	0.7267	ns
BSA+893 vs. PA	0.8779	ns
BSA+893 vs. PA+893	0.0994	ns
PA vs. PA+893	0.2729	ns
Figure 1O		
BSA vs. PAL	<0.0001	****
BSA vs. PAL VEC 893	0.731	ns

BSA vs. PAL TRPML 893	<0.0001	****
BSA vs. PAL GRP1DD 893	<0.0001	****
PAL vs. PAL VEC 893	<0.0001	****
PAL vs. PAL TRPML 893	0.0017	**
PAL vs. PAL GRP1DD 893	>0.9999	ns
PAL VEC 893 vs. PAL TRPML 893	<0.0001	****
PAL VEC 893 vs. PAL GRP1DD 893	<0.0001	****
PAL TRPML 893 vs. PAL GRP1DD 893	0.0231	*
Figure 1P		
BSA vs. PAL	<0.0001	****
BSA vs. PAL VEC 893	0.0869	ns
BSA vs. PAL TRPML 893	<0.0001	****
BSA vs. PAL GRP1DD 893	<0.0001	****
PAL vs. PAL VEC 893	<0.0001	****
PAL vs. PAL TRPML 893	0.0297	*
PAL vs. PAL GRP1DD 893	0.0015	**
PAL VEC 893 vs. PAL TRPML 893	<0.0001	****
PAL VEC 893 vs. PAL GRP1DD 893	<0.0001	****
PAL TRPML 893 vs. PAL GRP1DD 893	0.8915	ns
Figure 1Q		
BSA vs. PAL	<0.0001	****
BSA vs. PAL VEC 893	0.9964	ns
BSA vs. PAL TRPML 893	<0.0001	****
BSA vs. PAL GRP1DD 893	<0.0001	****
PAL vs. PAL VEC 893	<0.0001	****
PAL vs. PAL TRPML 893	0.0939	ns
PAL vs. PAL GRP1DD 893	0.3264	ns
PAL VEC 893 vs. PAL TRPML 893	<0.0001	****
PAL VEC 893 vs. PAL GRP1DD 893	<0.0001	****
PAL TRPML 893 vs. PAL GRP1DD 893	0.9727	ns
Figure 2B		
control vs. PAL	<0.0001	****
control vs. PAL:893	0.001	**
PAL vs. PAL:893	<0.0001	****
Figure 2D		
control vs. PAL	<0.0001	****
control vs. PAL:893	0.9019	ns
PAL vs. PAL:893	<0.0001	****
Figure 2E		
CHOP		
bsa vs. palmitate	<0.0001	****
bsa vs. bsa 893	0.9978	ns
bsa vs. pal 893	0.9269	ns
palmitate vs. bsa 893	<0.0001	****
palmitate vs. pal 893	<0.0001	****
bsa 893 vs. pal 893	0.972	ns
XBP1s		
bsa vs. palmitate	<0.0001	****
bsa vs. bsa 893	0.9932	ns
bsa vs. pal 893	0.9125	ns
palmitate vs. bsa 893	<0.0001	****

palmitate vs. pal 893<br>bsa 893 vs. pal 893	<0.0001<br>0.9787	****<br>ns
ATF6		
bsa vs. palmitate<br>bsa vs. bsa 893<br>bsa vs. pal 893<br>palmitate vs. bsa 893<br>palmitate vs. pal 893<br>bsa 893 vs. pal 893	0.0002<br>0.0964<br>0.0103<br><0.0001<br><0.0001<br>0.4171	***<br>ns<br>*<br>****<br>****<br>ns
GRP78		
bsa vs. palmitate<br>bsa vs. bsa 893<br>bsa vs. pal 893<br>palmitate vs. bsa 893<br>palmitate vs. pal 893<br>bsa 893 vs. pal 893	0.0022<br>0.7849<br>0.3266<br>0.0008<br>0.0003<br>0.8097	**<br>ns<br>ns<br>***<br>***<br>ns
Figure 3A		
SD veh vs. SD 893<br>SD veh vs. HFD veh<br>SD veh vs. HFD 893<br>SD 893 vs. HFD veh<br>SD 893 vs. HFD 893<br>HFD veh vs. HFD 893	0.617<br><0.0001<br><0.0001<br><0.0001<br><0.0001<br>0.9925	ns<br>****<br>****<br>****<br>****<br>ns
Figure 3C		
Vehicle:SD vs. 893:SD<br>Vehicle:SD vs. Vehicle:HFD<br>Vehicle:SD vs. 893:HFD<br>893:SD vs. Vehicle:HFD<br>893:SD vs. 893:HFD<br>Vehicle:HFD vs. 893:HFD	0.2201<br><0.0001<br>0.1839<br><0.0001<br>0.9997<br><0.0001	ns<br>****<br>ns<br>****<br>ns<br>****
Figure 3D		
Vehicle:SD vs. 893:SD<br>Vehicle:SD vs. Vehicle:HFD<br>Vehicle:SD vs. 893:HFD<br>893:SD vs. Vehicle:HFD<br>893:SD vs. 893:HFD<br>Vehicle:HFD vs. 893:HFD	0.7215<br><0.0001<br>0.421<br><0.0001<br>0.0522<br><0.0001	ns<br>****<br>ns<br>****<br>ns<br>****
Figure 3E		
Untreated SD vs. 120mpk 893 SD<br>Untreated SD vs. Untreated HFD<br>Untreated SD vs. 120mpk 893 HFD<br>120mpk 893 SD vs. Untreated HFD<br>120mpk 893 SD vs. 120mpk 893 HFD<br>Untreated HFD vs. 120mpk 893 HFD	0.9922<br><0.0001<br><0.0001<br><0.0001<br><0.0001<br>0.9986	ns<br>****<br>****<br>****<br>****<br>ns
Figure 3H		
Vehicle:SD vs. 893:SD<br>Vehicle:SD vs. Vehicle:HFD<br>Vehicle:SD vs. 893:HFD<br>893:SD vs. Vehicle:HFD<br>893:SD vs. 893:HFD<br>Vehicle:HFD vs. 893:HFD	0.0122<br><0.0001<br><0.0001<br><0.0001<br>0.0065<br><0.0001	*<br>****<br>****<br>****<br>**<br>****
Figure 3I		
Vehicle:SD vs. 893:SD<br>Vehicle:SD vs. Vehicle:HFD<br>Vehicle:SD vs. 893:HFD<br>893:SD vs. Vehicle:HFD<br>893:SD vs. 893:HFD<br>Vehicle:HFD vs. 893:HFD	0.0065<br><0.0001<br><0.0001<br><0.0001<br>0.2952<br><0.0001	**<br>****<br>****<br>****<br>ns<br>****
Figure 3K		
Vehicle:SD vs. 893:SD<br>Vehicle:SD vs. Vehicle:HFD<br>Vehicle:SD vs. 893:HFD	0.6348<br><0.0001<br>0.0033	ns<br>****<br>**

893:SD vs. Vehicle:HFD	<0.0001	****
893:SD vs. 893:HFD	0.0855	ns
Vehicle:HFD vs. 893:HFD	<0.0001	****
Figure 3L		
Vehicle:SD vs. 893:SD	0.144	ns
Vehicle:SD vs. Vehicle:HFD	<0.0001	****
Vehicle:SD vs. 893:HFD	0.9995	ns
893:SD vs. Vehicle:HFD	<0.0001	****
893:SD vs. 893:HFD	0.1149	ns
Vehicle:HFD vs. 893:HFD	<0.0001	****
Figure 4A		
SD veh vs. SD 893	0.9815	ns
SD veh vs. HFD veh	<0.0001	****
SD veh vs. HFD 893	<0.0001	****
SD 893 vs. HFD veh	<0.0001	****
SD 893 vs. HFD 893	<0.0001	****
HFD veh vs. HFD 893	>0.9999	ns
Figure 4C		
VEH:SD vs. 893:SD	<0.0001	****
VEH:SD vs. VEH:HFD	<0.0001	****
VEH:SD vs. 893:HFD	<0.0001	****
893:SD vs. VEH:HFD	<0.0001	****
893:SD vs. 893:HFD	0.9205	ns
VEH:HFD vs. 893:HFD	<0.0001	****
Figure 4D		
Untreated SD vs. 120mpk 893 SD	0.974	ns
Untreated SD vs. Untreated HFD	<0.0001	****
Untreated SD vs. 120mpk 893 HFD	<0.0001	****
120mpk 893 SD vs. Untreated HFD	<0.0001	****
120mpk 893 SD vs. 120mpk 893 HFD	<0.0001	****
Untreated HFD vs. 120mpk 893 HFD	>0.9999	ns
Figure 4F		
VEH:SD vs. 893:SD	0.005	**
VEH:SD vs. VEH:HFD	<0.0001	****
VEH:SD vs. 893:HFD	<0.0001	****
893:SD vs. VEH:HFD	<0.0001	****
893:SD vs. 893:HFD	0.0013	**
VEH:HFD vs. 893:HFD	<0.0001	****
Figure 4G		
VEH:SD vs. 893:SD	0.9057	ns
VEH:SD vs. VEH:HFD	<0.0001	****
VEH:SD vs. 893:HFD	<0.0001	****
893:SD vs. VEH:HFD	<0.0001	****
893:SD vs. 893:HFD	<0.0001	****
VEH:HFD vs. 893:HFD	0.8676	ns
Figure 4I		
VEH:SD vs. 893:SD	0.9998	ns
VEH:SD vs. VEH:HFD	<0.0001	****
VEH:SD vs. 893:HFD	<0.0001	****
893:SD vs. VEH:HFD	<0.0001	****
893:SD vs. 893:HFD	<0.0001	****
VEH:HFD vs. 893:HFD	<0.0001	****
Figure 4J		
VEH:SD vs. 893:SD	0.9342	ns
VEH:SD vs. VEH:HFD	<0.0001	****
VEH:SD vs. 893:HFD	<0.0001	****
893:SD vs. VEH:HFD	<0.0001	****
893:SD vs. 893:HFD	<0.0001	****
VEH:HFD vs. 893:HFD	0.7519	ns
Figure 4K		
CHOP	0.0401	*
XBP1s	0.0804	ns

ATF6<br>GRP78	0.057<br>0.0494	ns<br>*
Figure 5A		
SD vs. HFD vehicle<br>SD vs. HFD 893<br>SD vs. HFD pair-fed<br>HFD vehicle vs. HFD 893<br>HFD vehicle vs. HFD pair-fed<br>HFD 893 vs. HFD pair-fed	<0.0001<br><0.0001<br><0.0001<br>0.0006<br>0.0554<br><0.0001	****<br>****<br>****<br>***<br>ns<br>****
Figure 5B		
SD vs. HFD vehicle<br>SD vs. HFD 893<br>SD vs. HFD pair-fed<br>HFD vehicle vs. HFD 893<br>HFD vehicle vs. HFD pair-fed<br>HFD 893 vs. HFD pair-fed	0.0101<br>0.8744<br>0.0037<br>0.019<br>0.6772<br>0.0112	*<br>ns<br>**<br>*<br>ns<br>*
Figure 5C		
SD vs. HFD vehicle<br>SD vs. HFD 893<br>SD vs. HFD pair-fed<br>HFD vehicle vs. HFD 893<br>HFD vehicle vs. HFD pair-fed<br>HFD 893 vs. HFD pair-fed	0.002<br>0.0431<br>0.0017<br>0.0029<br>0.973<br>0.0035	**<br>*<br>**<br>**<br>ns<br>**
Figure 5D		
Rx 1<br>Rx 3<br>Rx 6<br>Rx 9	0.0024<br>0.0835<br>0.1403<br>0.3509	**<br>ns<br>ns<br>ns
Figure 5E		
Rx 1<br>Rx 3<br>Rx 6<br>Rx 9	0.0016<br>0.0006<br>0.0006<br>0.0008	**<br>***<br>***<br>***
Figure 5F		
Rx 1<br>Rx 3<br>Rx 6<br>Rx 9	0.0001<br>0.1042<br>0.0335<br>0.0065	***<br>ns<br>*<br>**
Figure 5G	0.0186	*
Figure 5H	0.0043	**
Figure 5J		
0<br>4<br>8<br>10<br>16	0.0164<br>0.0011<br>0.0029<br>0.0025<br>0.0001	*<br>***<br>**<br>**<br>***
Figure 5K		
0<br>4<br>8<br>10<br>16	0.1235<br>0.0000<br>0.0033<br>0.0136<br>0.0024	ns<br>***<br>**<br>*<br>**
Figure 5L		
0<br>4<br>8<br>10<br>16	0.1898<br>0.0004<br>0.0006<br>0.0019<br>0.0001	ns<br>***<br>***<br>**<br>***
Figure 6B		
1<br>2<br>3	0.0057<br>0.2985<br>0.0174	**<br>ns<br>*

4	0.5965	ns
Figure 6D		
1<br>2<br>3<br>4	0.0071<br>0.6322<br>0.0159<br>0.6017	**<br>ns<br>*<br>ns
Figure 6E		
vehicle vs. 893<br>vehicle vs. pair-fed<br>893 vs. pair-fed	<0.0001<br>0.0003<br>0.8575	****<br>***<br>ns
Figure 6F		
Day 49<br>SD:VEH vs. hfd:veh<br>SD:VEH vs. hfd:60mpk 893<br>SD:VEH vs. hfd:120 mpk 893<br>hfd:veh vs. hfd:60mpk 893<br>hfd:veh vs. hfd:120 mpk 893<br>hfd:60mpk 893 vs. hfd:120 mpk 893	 0.0003<br>0.0049<br>0.0045<br>0.8421<br>0.7943<br>0.9999	 ***<br>**<br>**<br>ns<br>ns<br>ns
Day 53<br>SD:VEH vs. hfd:veh<br>SD:VEH vs. hfd:60mpk 893<br>SD:VEH vs. hfd:120 mpk 893<br>hfd:veh vs. hfd:60mpk 893<br>hfd:veh vs. hfd:120 mpk 893<br>hfd:60mpk 893 vs. hfd:120 mpk 893	 0.0002<br>0.049<br>0.0757<br>0.2365<br>0.1307<br>0.9936	 ***<br>*<br>ns<br>ns<br>ns<br>ns
Day 60<br>SD:VEH vs. hfd:veh<br>SD:VEH vs. hfd:60mpk 893<br>SD:VEH vs. hfd:120 mpk 893<br>hfd:veh vs. hfd:60mpk 893<br>hfd:veh vs. hfd:120 mpk 893<br>hfd:60mpk 893 vs. hfd:120 mpk 893	 0.0002<br>0.1145<br>0.7592<br>0.1227<br>0.0036<br>0.5354	 ***<br>ns<br>ns<br>ns<br>**<br>ns
Day 67<br>SD:VEH vs. hfd:veh<br>SD:VEH vs. hfd:60mpk 893<br>SD:VEH vs. hfd:120 mpk 893<br>hfd:veh vs. hfd:60mpk 893<br>hfd:veh vs. hfd:120 mpk 893<br>hfd:60mpk 893 vs. hfd:120 mpk 893	 0.0001<br>0.1875<br>0.9769<br>0.0435<br>0.0004<br>0.3593	 ***<br>ns<br>ns<br>*<br>***<br>ns
Day 73<br>SD:VEH vs. hfd:veh<br>SD:VEH vs. hfd:60mpk 893<br>SD:VEH vs. hfd:120 mpk 893<br>hfd:veh vs. hfd:60mpk 893<br>hfd:veh vs. hfd:120 mpk 893<br>hfd:60mpk 893 vs. hfd:120 mpk 893	 0.0054<br>0.1853<br>>0.9999<br>0.2179<br>0.0064<br>0.25	 **<br>ns<br>ns<br>ns<br>**<br>ns
Figure 6G		
SD:VEH vs. HFD:VEH<br>SD:VEH vs. HFD:60 MPK 893<br>SD:VEH vs. HFD:120 MPK 893<br>HFD:VEH vs. HFD:60 MPK 893<br>HFD:VEH vs. HFD:120 MPK 893<br>HFD:60 MPK 893 vs. HFD:120 MPK 893	0.5691<br>0.0003<br><0.0001<br><0.0001<br><0.0001<br><0.0001	ns<br>***<br>****<br>****<br>****<br>****
Figure 6H		
Standard Diet vs. HFD + Vehicle<br>Standard Diet vs. HFD + 60 mpk 893<br>Standard Diet vs. HFD + 120 mpk 893<br>HFD + Vehicle vs. HFD + 60 mpk 893<br>HFD + Vehicle vs. HFD + 120 mpk 893<br>HFD + 60 mpk 893 vs. HFD + 120 mpk 893	<0.0001<br>0.1685<br>0.9016<br>0.0019<br><0.0001<br>0.4801	****<br>ns<br>ns<br>**<br>****<br>ns
Figure 6I		
SD:VEH vs. HFD:VEH<br>SD:VEH vs. HFD:60 MPK 893	0.3922<br>0.9851	ns<br>ns

SD:VEH vs. HFD:120 MPK 893	<0.0001	****
HFD:VEH vs. HFD:60 MPK 893	0.3448	ns
HFD:VEH vs. HFD:120 MPK 893	0.0001	***
HFD:60 MPK 893 vs. HFD:120 MPK 893	0.0062	**
Figure 6L	0.9521	ns
Figure 6J		
Standard Diet vs. HFD + Vehicle	0.6327	ns
Standard Diet vs. HFD + 60 mpk 893	0.8207	ns
Standard Diet vs. HFD + 120 mpk 893	0.8397	ns
HFD + Vehicle vs. HFD + 60 mpk 893	0.9908	ns
HFD + Vehicle vs. HFD + 120 mpk 893	0.1975	ns
HFD + 60 mpk 893 vs. HFD + 120 mpk 893	0.3506	ns
Figure 6K		
SD:VEH vs. HFD:VEH	0.7292	ns
SD:VEH vs. HFD:60 MPK 893	<0.0001	****
SD:VEH vs. HFD:120 MPK 893	<0.0001	****
HFD:VEH vs. HFD:60 MPK 893	0.0006	***
HFD:VEH vs. HFD:120 MPK 893	0.0001	***
HFD:60 MPK 893 vs. HFD:120 MPK 893	0.9671	ns
Figure 6M		
SD vs. veh hfd	0.6012	ns
SD vs. 893 hfd	<0.0001	****
SD vs. wheel hfd	<0.0001	****
SD vs. wheel 893 hfd	<0.0001	****
veh hfd vs. 893 hfd	<0.0001	****
veh hfd vs. wheel hfd	<0.0001	****
veh hfd vs. wheel 893 hfd	<0.0001	****
893 hfd vs. wheel hfd	0.008	**
893 hfd vs. wheel 893 hfd	<0.0001	****
wheel hfd vs. wheel 893 hfd	<0.0001	****
Figure 6N		
SD vs. HFD + Vehicle	<0.0001	****
SD vs. HFD + 120 mpk 893	0.958	ns
SD vs. HFD + Vehicle + Wheel	0.1533	ns
SD vs. HFD + 120 mpk 893 + Wheel	0.9331	ns
HFD + Vehicle vs. HFD + 120 mpk 893	<0.0001	****
HFD + Vehicle vs. HFD + Vehicle + Wheel	0.0071	**
HFD + Vehicle vs. HFD + 120 mpk 893 + Wheel	<0.0001	****
HFD + 120 mpk 893 vs. HFD + Vehicle + Wheel	0.4489	ns
HFD + 120 mpk 893 vs. HFD + 120 mpk 893 + Wheel	0.6427	ns
HFD + Vehicle + Wheel vs. HFD + 120 mpk 893 + Wheel	0.0562	ns
Figure 6O		
SD vs. SD 893	0.0076	**
SD vs. HFD VEH	<0.0001	****
SD vs. HFD 893	0.2512	ns
SD 893 vs. HFD VEH	<0.0001	****
SD 893 vs. HFD 893	<0.0001	****
HFD VEH vs. HFD 893	<0.0001	****
Figure 6P		
SD vs. SD 893	0.0075	**
SD vs. HFD VEH	>0.9999	ns
SD vs. HFD 893	<0.0001	****
SD 893 vs. HFD VEH	0.0075	**
SD 893 vs. HFD 893	0.0641	ns
HFD VEH vs. HFD 893	<0.0001	****
Figure 6R		
SD vs. SD 893	0.0248	*
SD vs. HFD VEH	0.9981	ns
SD vs. HFD 893	<0.0001	****
SD 893 vs. HFD VEH	0.0162	*
SD 893 vs. HFD 893	<0.0001	****
HFD VEH vs. HFD 893	<0.0001	****
Figure 7C		

d17:1/22:0<br>sd vs. hfd<br>sd vs. hfd 893<br>hfd vs. hfd 893	 0.0628<br>0.0324<br>0.9065	 ns<br>*<br>ns
d18:1/16:0<br>sd vs. hfd<br>sd vs. hfd 893<br>hfd vs. hfd 893	 0.0635<br>0.6841<br>0.017	 ns<br>ns<br>*
d18:1/18:0<br>sd vs. hfd<br>sd vs. hfd 893<br>hfd vs. hfd 893	 0.5271<br>0.7527<br>0.2089	 ns<br>ns<br>ns
d18:1/20:0<br>sd vs. hfd<br>sd vs. hfd 893<br>hfd vs. hfd 893	 0.0033<br>0.4125<br>0.024	 **<br>ns<br>*
d18:1/22:0<br>sd vs. hfd<br>sd vs. hfd 893<br>hfd vs. hfd 893	 0.1027<br>0.2328<br>0.8464	 ns<br>ns<br>ns
d18:1/22:1<br>sd vs. hfd<br>sd vs. hfd 893<br>hfd vs. hfd 893	 0.4812<br>0.4557<br>0.0848	 ns<br>ns<br>ns
d18:1/23:0<br>sd vs. hfd<br>sd vs. hfd 893<br>hfd vs. hfd 893	 0.3986<br>0.513<br>0.974	 ns<br>ns<br>ns
d18:1/24:0<br>sd vs. hfd<br>sd vs. hfd 893<br>hfd vs. hfd 893	 0.7884<br>0.9571<br>0.923	 ns<br>ns<br>ns
d18:1/24:1<br>sd vs. hfd<br>sd vs. hfd 893<br>hfd vs. hfd 893	 0.7359<br>0.888<br>0.9544	 ns<br>ns<br>ns
d18:2/18:0<br>sd vs. hfd<br>sd vs. hfd 893<br>hfd vs. hfd 893	 0.9577<br>0.7692<br>0.6061	 ns<br>ns<br>ns
d18:2/22:0<br>sd vs. hfd<br>sd vs. hfd 893<br>hfd vs. hfd 893	 0.098<br>0.6517<br>0.3561	 ns<br>ns<br>ns
d18:2/24:1<br>sd vs. hfd<br>sd vs. hfd 893<br>hfd vs. hfd 893	 0.2949<br>0.9964<br>0.3278	 ns<br>ns<br>ns
Figure 7D		
SD vs. HFD + vehicle<br>SD vs. HFD + 893<br>HFD + vehicle vs. HFD + 893	0.0863<br>0.9436<br>0.0521	ns<br>ns<br>ns
Figure 7E		
SD vs. HFD Vehicle<br>SD vs. HFD 60mpk 893<br>SD vs. HFD 120 mpk 893<br>HFD Vehicle h vs. HFD 60mpk 893<br>HFD Vehicle vs. HFD 120 mpk 893	0.0006<br>0.0757<br>>0.9999<br>0.2574<br>0.0018	***<br>ns<br>ns<br>ns<br>**
Figure 7G		
SD vs. HFD + Vehicle<br>SD vs. HFD + 60 mg/kg 893<br>SD vs. HFD + 120 mg/kg 893 - Trough<br>HFD + Vehicle vs. HFD + 60 mg/kg 893<br>HFD + Vehicle vs. HFD + 120 mg/kg 893 - Trough	0.0055<br>0.9882<br>0.9941<br>0.0244<br>0.0213	**<br>ns<br>ns<br>*<br>*

HFD + 60 mg/kg 893 vs. HFD + 120 mg/kg 893 - Trough	>0.9999	ns
Figure 7H		
SD vs. HFD Rx1 VEH	0.0861	ns
SD vs. HFD Rx1 893	0.9849	ns
SD vs. HFD Rx9 VEH	0.0007	***
SD vs. HFD Rx9 893	0.1497	ns
HFD Rx1 VEH vs. HFD Rx1 893	0.2374	ns
HFD Rx1 VEH vs. HFD Rx9 VEH	0.3787	ns
HFD Rx1 VEH vs. HFD Rx9 893	0.0002	***
HFD Rx1 893 vs. HFD Rx9 VEH	0.0033	**
HFD Rx1 893 vs. HFD Rx9 893	0.0492	*
HFD Rx9 VEH vs. HFD Rx9 893	<0.0001	****
Figure 7I		
sd vs. HFD vehicle	0.3388	ns
sd vs. HFD 893	0.0029	**
sd vs. HFD pair-fed	0.0021	**
HFD vehicle vs. HFD 893	0.0337	*
HFD vehicle vs. HFD pair-fed	0.0299	*
HFD 893 vs. HFD pair-fed	0.9994	ns
Figure EV2B		
ETOH vs. C16-CER	<0.0001	****
ETOH vs. ETOH+Mdivi1+M1 1 h	0.3836	ns
ETOH vs. CER:MdiviM1 1 h	<0.0001	****
ETOH vs. ETOH:Mdivi1+M1 24 h	>0.9999	ns
ETOH vs. CER:Mdivi1+M1 24 h	<0.0001	****
C16-CER vs. ETOH+Mdivi1+M1 1 h	<0.0001	****
C16-CER vs. CER:MdiviM1 1 h	0.9072	ns
C16-CER vs. ETOH:Mdivi1+M1 24 h	<0.0001	****
C16-CER vs. CER:Mdivi1+M1 24 h	>0.9999	ns
ETOH+Mdivi1+M1 1 h vs. CER:MdiviM1 1 h	<0.0001	****
ETOH+Mdivi1+M1 1 h vs. ETOH:Mdivi1+M1 24 h	0.2996	ns
ETOH+Mdivi1+M1 1 h vs. CER:Mdivi1+M1 24 h	<0.0001	****
CER:MdiviM1 1 h vs. ETOH:Mdivi1+M1 24 h	<0.0001	****
CER:MdiviM1 1 h vs. CER:Mdivi1+M1 24 h	0.9113	ns
ETOH:Mdivi1+M1 24 h vs. CER:Mdivi1+M1 24 h	<0.0001	****
Figure EV2C		
ETOH vs. C16-CER	<0.0001	****
ETOH vs. ETOH+Mdivi1+M1 1 h	0.1119	ns
ETOH vs. CER:MdiviM1 1 h	<0.0001	****
ETOH vs. ETOH:Mdivi1+M1 24 h	0.4579	ns
ETOH vs. CER:Mdivi1+M1 24 h	<0.0001	****
C16-CER vs. ETOH+Mdivi1+M1 1 h	<0.0001	****
C16-CER vs. CER:MdiviM1 1 h	0.0902	ns
C16-CER vs. ETOH:Mdivi1+M1 24 h	<0.0001	****
C16-CER vs. CER:Mdivi1+M1 24 h	0.0062	**
ETOH+Mdivi1+M1 1 h vs. CER:MdiviM1 1 h	<0.0001	****
ETOH+Mdivi1+M1 1 h vs. ETOH:Mdivi1+M1 24 h	0.9946	ns
ETOH+Mdivi1+M1 1 h vs. CER:Mdivi1+M1 24 h	<0.0001	****
CER:MdiviM1 1 h vs. ETOH:Mdivi1+M1 24 h	<0.0001	****
CER:MdiviM1 1 h vs. CER:Mdivi1+M1 24 h	0.8549	ns
ETOH:Mdivi1+M1 24 h vs. CER:Mdivi1+M1 24 h	<0.0001	****
Figure EV2D		
ETOH vs. C16-CER	<0.0001	****
ETOH vs. ETOH+Mdivi1+M1 1 h	0.0898	ns
ETOH vs. CER:MdiviM1 1 h	<0.0001	****
ETOH vs. ETOH:Mdivi1+M1 24 h	0.0116	*
ETOH vs. CER:Mdivi1+M1 24 h	<0.0001	****
C16-CER vs. ETOH+Mdivi1+M1 1 h	<0.0001	****
C16-CER vs. CER:MdiviM1 1 h	>0.9999	ns
C16-CER vs. ETOH:Mdivi1+M1 24 h	<0.0001	****
C16-CER vs. CER:Mdivi1+M1 24 h	0.9696	ns
ETOH+Mdivi1+M1 1 h vs. CER:MdiviM1 1 h	<0.0001	****
ETOH+Mdivi1+M1 1 h vs. ETOH:Mdivi1+M1 24 h	<0.0001	****
ETOH+Mdivi1+M1 1 h vs. CER:Mdivi1+M1 24 h	<0.0001	****

CER:MdiviM1 1 h vs. ETOH:Mdivi1+M1 24 h	<0.0001	****
CER:MdiviM1 1 h vs. CER:Mdivi1+M1 24 h	0.9935	ns
ETOH:Mdivi1+M1 24 h vs. CER:Mdivi1+M1 24 h	<0.0001	****
Figure EV2F		
ETOH vs. C16CER	<0.0001	****
ETOH vs. ETOH:Lef 1 h	0.5292	ns
ETOH vs. CER:LEF 1 H	<0.0001	****
ETOH vs. ETOH:Lef 24 h	0.9941	ns
ETOH vs. CER:Lef 24 H	0.1068	ns
C16CER vs. ETOH:Lef 1 h	<0.0001	****
C16CER vs. CER:LEF 1 H	>0.9999	ns
C16CER vs. ETOH:Lef 24 h	<0.0001	****
C16CER vs. CER:Lef 24 H	<0.0001	****
ETOH:Lef 1 h vs. CER:LEF 1 H	<0.0001	****
ETOH:Lef 1 h vs. ETOH:Lef 24 h	0.9734	ns
ETOH:Lef 1 h vs. CER:Lef 24 H	0.9989	ns
CER:LEF 1 H vs. ETOH:Lef 24 h	<0.0001	****
CER:LEF 1 H vs. CER:Lef 24 H	<0.0001	****
ETOH:Lef 24 h vs. CER:Lef 24 H	0.4144	ns
Figure EV2G		
ETOH vs. C16CER	<0.0001	****
ETOH vs. ETOH:Lef 1 h	0.7502	ns
ETOH vs. CER:LEF 1 H	<0.0001	****
ETOH vs. ETOH:Lef 24 h	0.0063	**
ETOH vs. CER:Lef 24 H	0.0562	ns
C16CER vs. ETOH:Lef 1 h	<0.0001	****
C16CER vs. CER:LEF 1 H	0.9806	ns
C16CER vs. ETOH:Lef 24 h	<0.0001	****
C16CER vs. CER:Lef 24 H	<0.0001	****
ETOH:Lef 1 h vs. CER:LEF 1 H	<0.0001	****
ETOH:Lef 1 h vs. ETOH:Lef 24 h	0.2495	ns
ETOH:Lef 1 h vs. CER:Lef 24 H	0.6842	ns
CER:LEF 1 H vs. ETOH:Lef 24 h	<0.0001	****
CER:LEF 1 H vs. CER:Lef 24 H	<0.0001	****
ETOH:Lef 24 h vs. CER:Lef 24 H	0.9801	ns
Figure EV2H		
ETOH vs. C16CER	<0.0001	****
ETOH vs. ETOH:Lef 1 h	>0.9999	ns
ETOH vs. CER:LEF 1 H	<0.0001	****
ETOH vs. ETOH:Lef 24 h	>0.9999	ns
ETOH vs. CER:Lef 24 H	0.9398	ns
C16CER vs. ETOH:Lef 1 h	<0.0001	****
C16CER vs. CER:LEF 1 H	>0.9999	ns
C16CER vs. ETOH:Lef 24 h	<0.0001	****
C16CER vs. CER:Lef 24 H	<0.0001	****
ETOH:Lef 1 h vs. CER:LEF 1 H	<0.0001	****
ETOH:Lef 1 h vs. ETOH:Lef 24 h	>0.9999	ns
ETOH:Lef 1 h vs. CER:Lef 24 H	0.888	ns
CER:LEF 1 H vs. ETOH:Lef 24 h	<0.0001	****
CER:LEF 1 H vs. CER:Lef 24 H	<0.0001	****
ETOH:Lef 24 h vs. CER:Lef 24 H	0.8578	ns
Figure EV2J		
ETOH vs. C16-CER	<0.0001	****
ETOH vs. ETOH:P110 1 h at 10 uM	0.9974	ns
ETOH vs. CER:P110 10 UM 1 H	<0.0001	****
ETOH vs. ETOH:P110 12 h	0.8708	ns
ETOH vs. CER:P110 12h	0.0004	***
C16-CER vs. ETOH:P110 1 h at 10 uM	<0.0001	****
C16-CER vs. CER:P110 10 UM 1 H	0.2168	ns
C16-CER vs. ETOH:P110 12 h	<0.0001	****
C16-CER vs. CER:P110 12h	<0.0001	****
ETOH:P110 1 h at 10 uM vs. CER:P110 10 UM 1 H	<0.0001	****
ETOH:P110 1 h at 10 uM vs. ETOH:P110 12 h	0.9999	ns
ETOH:P110 1 h at 10 uM vs. CER:P110 12h	0.0001	***

CER:P110 10 UM 1 H vs. ETOH:P110 12 h	<0.0001	****
CER:P110 10 UM 1 H vs. CER:P110 12h	<0.0001	****
ETOH:P110 12 h vs. CER:P110 12h	0.0014	**
Figure EV2K		
ETOH vs. C16-CER	<0.0001	****
ETOH vs. ETOH:P110 1 h at 10 uM	>0.9999	ns
ETOH vs. CER:P110 10 UM 1 H	<0.0001	****
ETOH vs. ETOH:P110 12 h	0.8591	ns
ETOH vs. CER:P110 12h	>0.9999	ns
C16-CER vs. ETOH:P110 1 h at 10 uM	<0.0001	****
C16-CER vs. CER:P110 10 UM 1 H	0.6603	ns
C16-CER vs. ETOH:P110 12 h	<0.0001	****
C16-CER vs. CER:P110 12h	<0.0001	****
ETOH:P110 1 h at 10 uM vs. CER:P110 10 UM 1 H	<0.0001	****
ETOH:P110 1 h at 10 uM vs. ETOH:P110 12 h	0.9729	ns
ETOH:P110 1 h at 10 uM vs. CER:P110 12h	>0.9999	ns
CER:P110 10 UM 1 H vs. ETOH:P110 12 h	<0.0001	****
CER:P110 10 UM 1 H vs. CER:P110 12h	<0.0001	****
ETOH:P110 12 h vs. CER:P110 12h	0.8473	ns
Figure EV2L		
ETOH vs. C16-CER	<0.0001	****
ETOH vs. ETOH:P110 1 h at 10 uM	0.3298	ns
ETOH vs. CER:P110 10 UM 1 H	<0.0001	****
ETOH vs. ETOH:P110 12 h	0.7725	ns
ETOH vs. CER:P110 12h	0.5592	ns
C16-CER vs. ETOH:P110 1 h at 10 uM	<0.0001	****
C16-CER vs. CER:P110 10 UM 1 H	0.0506	ns
C16-CER vs. ETOH:P110 12 h	<0.0001	****
C16-CER vs. CER:P110 12h	<0.0001	****
ETOH:P110 1 h at 10 uM vs. CER:P110 10 UM 1 H	<0.0001	****
ETOH:P110 1 h at 10 uM vs. ETOH:P110 12 h	0.9812	ns
ETOH:P110 1 h at 10 uM vs. CER:P110 12h	0.9991	ns
CER:P110 10 UM 1 H vs. ETOH:P110 12 h	<0.0001	****
CER:P110 10 UM 1 H vs. CER:P110 12h	<0.0001	****
ETOH:P110 12 h vs. CER:P110 12h	0.9994	ns
Figure EV2N		
BSA vs. PAL	<0.0001	****
BSA vs. BSA:WFA	>0.9999	ns
BSA vs. PAL:WFA	<0.0001	****
BSA vs. BSA:CEL	<0.0001	****
BSA vs. PAL:CEL	<0.0001	****
PAL vs. BSA:WFA	<0.0001	****
PAL vs. PAL:WFA	0.9963	ns
PAL vs. BSA:CEL	0.9178	ns
PAL vs. PAL:CEL	0.0651	ns
BSA:WFA vs. PAL:WFA	<0.0001	****
BSA:WFA vs. BSA:CEL	<0.0001	****
BSA:WFA vs. PAL:CEL	<0.0001	****
PAL:WFA vs. BSA:CEL	>0.9999	ns
PAL:WFA vs. PAL:CEL	0.4601	ns
BSA:CEL vs. PAL:CEL	0.9545	ns
Figure EV2O		
BSA vs. PAL	<0.0001	****
BSA vs. BSA:WFA	0.8747	ns
BSA vs. PAL:WFA	<0.0001	****
BSA vs. BSA:CEL	<0.0001	****
BSA vs. PAL:CEL	<0.0001	****
PAL vs. BSA:WFA	<0.0001	****
PAL vs. PAL:WFA	<0.0001	****
PAL vs. BSA:CEL	0.0634	ns
PAL vs. PAL:CEL	0.014	*
BSA:WFA vs. PAL:WFA	<0.0001	****
BSA:WFA vs. BSA:CEL	<0.0001	****
BSA:WFA vs. PAL:CEL	<0.0001	****

PAL:WFA vs. BSA:CEL<br>PAL:WFA vs. PAL:CEL<br>BSA:CEL vs. PAL:CEL	0.3491<br>0.4251<br>>0.9999	ns<br>ns<br>ns
Figure EV2P		
BSA vs. PAL<br>BSA vs. BSA:WFA<br>BSA vs. PAL:WFA<br>BSA vs. BSA:CEL<br>BSA vs. PAL:CEL<br>PAL vs. BSA:WFA<br>PAL vs. PAL:WFA<br>PAL vs. BSA:CEL<br>PAL vs. PAL:CEL<br>BSA:WFA vs. PAL:WFA<br>BSA:WFA vs. BSA:CEL<br>BSA:WFA vs. PAL:CEL<br>PAL:WFA vs. BSA:CEL<br>PAL:WFA vs. PAL:CEL<br>BSA:CEL vs. PAL:CEL	<0.0001<br>0.0335<br><0.0001<br><0.0001<br><0.0001<br><0.0001<br>0.1968<br>0.3089<br>0.0201<br><0.0001<br><0.0001<br><0.0001<br>0.9999<br>0.9503<br>0.8724	****<br>*<br>****<br>****<br>****<br>****<br>ns<br>ns<br>*<br>****<br>****<br>****<br>ns<br>ns<br>ns
Figure EV2R		
DMSO vs. FCCP<br>DMSO vs. DMSO:893<br>DMSO vs. FCCP:893<br>FCCP vs. DMSO:893<br>FCCP vs. FCCP:893<br>DMSO:893 vs. FCCP:893	<0.0001<br>0.408<br><0.0001<br><0.0001<br>0.2613<br><0.0001	****<br>ns<br>****<br>****<br>ns<br>****
Figure EV2S		
DMSO vs. FCCP<br>DMSO vs. DMSO:893<br>DMSO vs. FCCP:893<br>FCCP vs. DMSO:893<br>FCCP vs. FCCP:893<br>DMSO:893 vs. FCCP:893	<0.0001<br>0.9997<br><0.0001<br><0.0001<br>0.3234<br><0.0001	****<br>ns<br>****<br>****<br>ns<br>****
Figure EV2T		
DMSO vs. FCCP<br>DMSO vs. DMSO:893<br>DMSO vs. FCCP:893<br>FCCP vs. DMSO:893<br>FCCP vs. FCCP:893<br>DMSO:893 vs. FCCP:893	<0.0001<br>0.4076<br><0.0001<br><0.0001<br>0.9125<br><0.0001	****<br>ns<br>****<br>****<br>ns<br>****
Figure EV4A		
1<br>2<br>3<br>4	0.1513<br>0.9172<br>0.2733<br>0.5713	ns<br>ns<br>ns<br>ns
Figure EV4B		
1<br>2<br>3<br>4	0.1411<br>0.6381<br>0.0013<br>0.8549	ns<br>ns<br>**<br>ns
Figure EV4E	0.8596	ns
Figure EV4F		
SD vs. HFD:VEH<br>SD vs. HFD:893<br>SD vs. HFD:VEH:WHEEL<br>SD vs. HFD:893:WHEEL<br>HFD:VEH vs. HFD:893<br>HFD:VEH vs. HFD:VEH:WHEEL<br>HFD:VEH vs. HFD:893:WHEEL<br>HFD:893 vs. HFD:VEH:WHEEL<br>HFD:893 vs. HFD:893:WHEEL<br>HFD:VEH:WHEEL vs. HFD:893:WHEEL	0.225<br><0.0001<br>0.0286<br><0.0001<br><0.0001<br><0.0001<br><0.0001<br>0.1764<br>0.0176<br><0.0001	ns<br>****<br>*<br>****<br>****<br>****<br>****<br>ns<br>*<br>****

Figure EV4G		
Standard Diet vs. HFD + Vehicle<br>Standard Diet vs. HFD + 120 mpk 893<br>Standard Diet vs. HFD + Vehicle + Wheel<br>Standard Diet vs. HFD + 120 mpk 893 + Wheel<br>HFD + Vehicle vs. HFD + 120 mpk 893<br>HFD + Vehicle vs. HFD + Vehicle + Wheel<br>HFD + Vehicle vs. HFD + 120 mpk 893 + Wheel<br>HFD + 120 mpk 893 vs. HFD + Vehicle + Wheel<br>HFD + 120 mpk 893 vs. HFD + 120 mpk 893 + Wheel<br>HFD + Vehicle + Wheel vs. HFD + 120 mpk 893 + Wheel	<0.0001<br>0.9516<br>0.2118<br>0.8166<br><0.0001<br>0.0027<br><0.0001<br>0.5707<br>0.4469<br>0.0415	****<br>ns<br>ns<br>ns<br>****<br>**<br>****<br>ns<br>ns<br>*
Figure EV4H		
SD vs. HFD:VEH<br>SD vs. HFD:893<br>SD vs. HFD:VEH:WHEEL<br>SD vs. HFD:893:WHEEL<br>HFD:VEH vs. HFD:893<br>HFD:VEH vs. HFD:VEH:WHEEL<br>HFD:VEH vs. HFD:893:WHEEL<br>HFD:893 vs. HFD:VEH:WHEEL<br>HFD:893 vs. HFD:893:WHEEL<br>HFD:VEH:WHEEL vs. HFD:893:WHEEL	0.8945<br><0.0001<br>0.0097<br><0.0001<br>0.001<br>0.08<br><0.0001<br>0.6315<br>0.3563<br>0.0351	ns<br>****<br>**<br>****<br>***<br>ns<br>****<br>ns<br>ns<br>*
Figure EV4I		
SD vs. HFD:VEH<br>SD vs. HFD:893<br>SD vs. HFD:VEH:WHEEL<br>SD vs. HFD:893:WHEEL<br>HFD:VEH vs. HFD:893<br>HFD:VEH vs. HFD:VEH:WHEEL<br>HFD:VEH vs. HFD:893:WHEEL<br>HFD:893 vs. HFD:VEH:WHEEL<br>HFD:893 vs. HFD:893:WHEEL<br>HFD:VEH:WHEEL vs. HFD:893:WHEEL	0.6908<br>0.8936<br>0.9952<br>0.9258<br>0.1984<br>0.9149<br>0.3144<br>0.7303<br>>0.9999<br>0.7989	ns<br>ns<br>ns<br>ns<br>ns<br>ns<br>ns<br>ns<br>ns<br>ns
Figure EV5A		
d17:1/22:0<br>sd vs. hfd<br>sd vs. hfd 893<br>hfd vs. hfd 893	 0.9991<br>0.3733<br>0.3921	 ns<br>ns<br>ns
d18:1/16:0<br>sd vs. hfd<br>sd vs. hfd 893<br>hfd vs. hfd 893	 0.5856<br>0.1777<br>0.6277	 ns<br>ns<br>ns
d18:1/18:0<br>sd vs. hfd<br>sd vs. hfd 893<br>hfd vs. hfd 893	 0.4583<br>0.8782<br>0.4694	 ns<br>ns<br>ns
d18:1/20:0<br>sd vs. hfd<br>sd vs. hfd 893<br>hfd vs. hfd 893	 0.9985<br>0.3002<br>0.3212	 ns<br>ns<br>ns
d18:1/22:0<br>sd vs. hfd<br>sd vs. hfd 893<br>hfd vs. hfd 893	 0.9945<br>0.3381<br>0.3835	 ns<br>ns<br>ns
d18:1/22:1<br>sd vs. hfd<br>sd vs. hfd 893<br>hfd vs. hfd 893	 0.9479<br>0.3291<br>0.4808	 ns<br>ns<br>ns
d18:1/23:0<br>sd vs. hfd<br>sd vs. hfd 893<br>hfd vs. hfd 893	 0.9665<br>0.1742<br>0.2483	 ns<br>ns<br>ns
d18:1/24:0<br>sd vs. hfd	 0.9907	 ns

sd vs. hfd 893<br>hfd vs. hfd 893	0.2412<br>0.2881	ns<br>ns
d18:1/24:1<br>sd vs. hfd<br>sd vs. hfd 893<br>hfd vs. hfd 893	 0.9854<br>0.2049<br>0.2577	 ns<br>ns<br>ns
d18:2/18:0<br>sd vs. hfd<br>sd vs. hfd 893<br>hfd vs. hfd 893	 0.3536<br>0.7674<br>0.1317	 ns<br>ns<br>ns
d18:2/22:0<br>sd vs. hfd<br>sd vs. hfd 893<br>hfd vs. hfd 893	 0.8879<br>0.3194<br>0.5542	 ns<br>ns<br>ns
d18:2/24:1<br>sd vs. hfd<br>sd vs. hfd 893<br>hfd vs. hfd 893	 0.795<br>0.2207<br>0.5062	 ns<br>ns<br>ns
