# Supplementary material for: Drug‐like sphingolipid SH‐BC‐893 opposes ceramide‐induced mitochondrial fission and corrects diet‐induced obesity
Source: EMBO Mol Med. 2021 Jul 7;13(8):e13086. doi: 10.15252/emmm.202013086 (PMC8350895; doi:10.15252/emmm.202013086)
Supplement: Supplementary file 2 — Expanded View Figures PDF [file EMMM-13-e13086-s002.pdf]

Expanded View Figures

Figure EV1. Strategy for morphometric analysis of mitochondrial networks in vitro.

Representative images of citrate synthase staining in MEFs treated with vehicle (left panels) or palmitate (PA, right panels). Images are maximum intensity Z-projections derived from 8 Z-slices. Binarized mitochondrial networks were segmented to tag individual objects. Aspect ratio (tubule width/length) as well as roundness ((4 × area)/(π × width)) were measured for all citrate synthase-positive objects on a per cell basis. Skeletonized networks were used to quantify branch length of the tubules. Violin plots show all citrate positive objects in the representative cell (left); the center line is the median and the quartiles define the 25th to 75th percentile. The scatter plots show mean ± SD from 40 cells from 2 biological replicates. Scale bar, 20 μm.

© 2021 The Authors

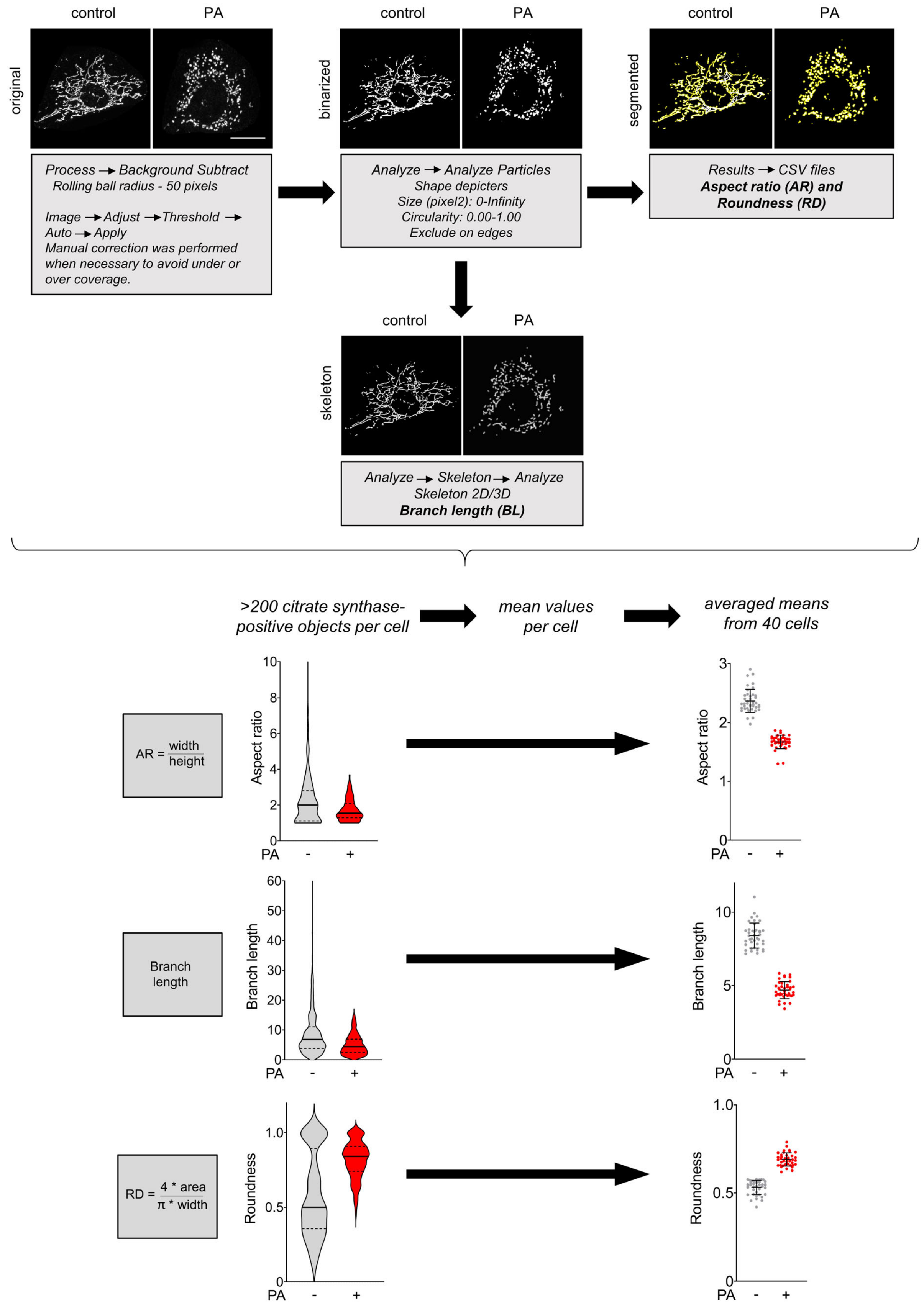
control
PA
original
binarized
segmented
skeleton
Process → Background Subtract
Rolling ball radius - 50 pixels
Image → Adjust → Threshold →
Auto → Apply
Manual correction was performed when necessary to avoid under or over coverage.
Analyze → Analyze Particles
Shape depicters
Size (pixel2): 0-Infinity
Circularity: 0.00-1.00
Exclude on edges
Results → CSV files
Aspect ratio (AR) and Roundness (RD)
Analyze → Skeleton → Analyze Skeleton 2D/3D
Branch length (BL)
>200 citrate synthase-positive objects per cell
mean values per cell
averaged means from 40 cells
AR = width / height
Branch length
RD = 4 * area / π * width
Aspect ratio
Branch length
Roundness
PA
-
+

Figure EV1.

Figure EV2. Existing agents do not protect from ceramide-induced mitochondrial fission.

A After a 1-h pre-treatment with vehicle (DMSO) or mdivi-1 and M1 (50 μM, 5 μM) for 1 h or 24 h, MEFs were treated for 3 h with vehicle (ethanol) or C16-CER (100 μM) and stained for citrate synthase.
B–D Aspect ratio (B), branch length (C), or roundness (D) of mitochondria in the cells in (A).
E–H As in (A-D), but in MEFs pre-treated with vehicle (methanol) or leflunomide (50 μM) for 1 h or 24 h.
I–L As in (A-D), but in MEFs pre-treated with vehicle (water) or P110 (10 μM) for 1 h or P110 (1 μM) for 12 h.
M MEFs treated for 3 h with BSA or palmitate (250 μM) after a 3-h pre-treatment with vehicle (DMSO), celastrol (500 nM), or withaferin A (WFA, 500 nM).
N–P Aspect ratio (N), branch length (O), or roundness (P) of mitochondria in the cells in (M).
Q MEFs were pre-treated with vehicle (water) or SH-BC-893 for 3 h and then treated with vehicle (DMSO) or FCCP (1 μM) for 1 h.
R–T Aspect ratio (R), branch length (S), or roundness (T) of mitochondria in the cells in (Q).

Data information: In B-D, F-H, J-L, N-P, and R-T, 40 cells from 2 biological replicates were evaluated and mean ± SD shown. Using a one-way ANOVA with Tukey's correction (D, G, H, P, and T) or Brown–Forsythe and Welch ANOVA tests with Dunnett's correction for multiple comparisons (B, C, F, J-L, N, O, R, and S), ***$P \leq 0.001$; **$P \leq 0.01$; *$P \leq 0.05$; ns, not significant, $P > 0.05$. Scale bars, 20 μm.

© 2021 The Authors

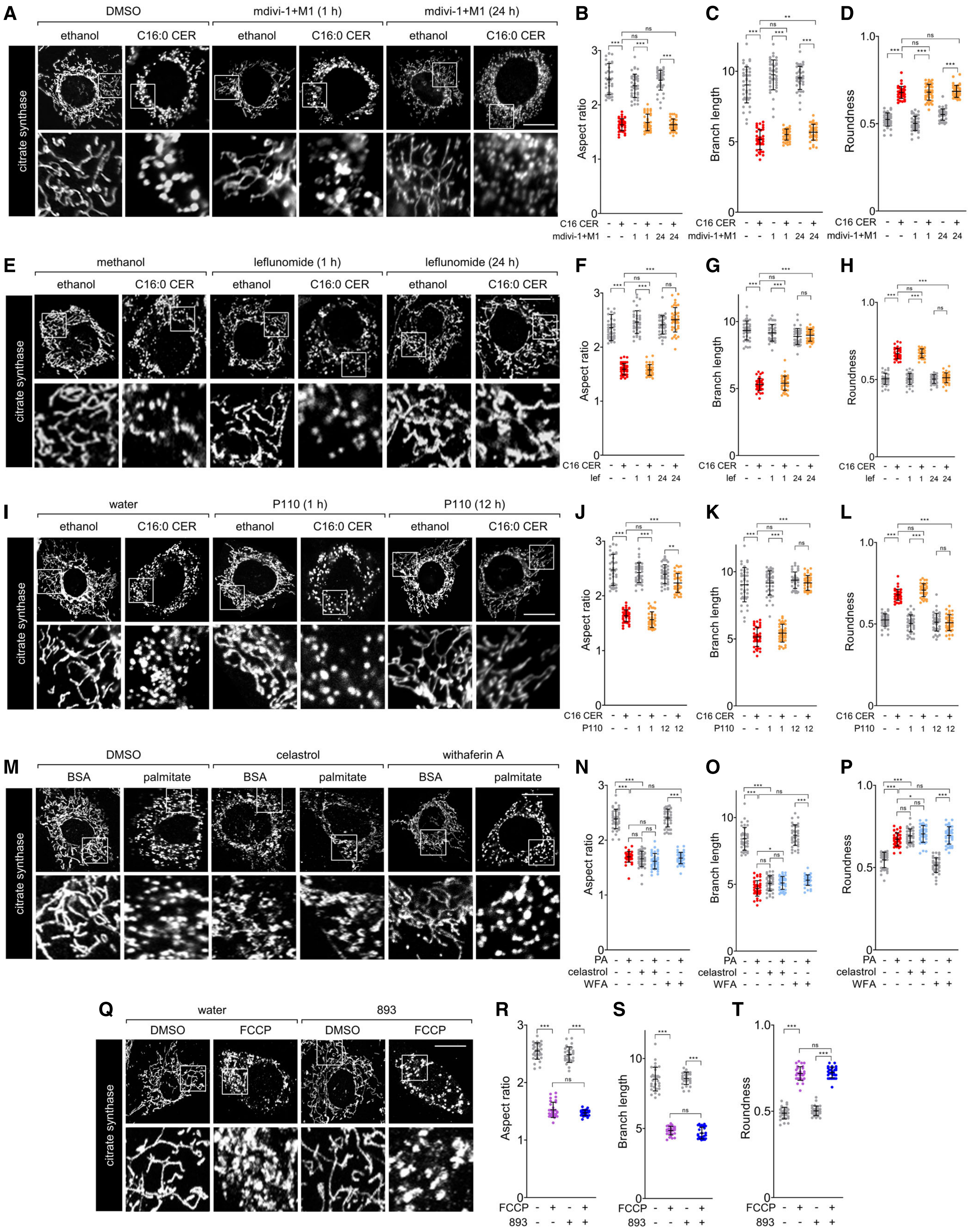
A
DMSO
mdivi-1+M1 (1 h)
mdivi-1+M1 (24 h)
ethanol
C16:0 CER
citrate synthase
B
C
D
Aspect ratio
Branch length
Roundness
C16 CER
mdivi-1+M1
E
methanol
leflunomide (1 h)
leflunomide (24 h)
F
G
H
lef
I
water
P110 (1 h)
P110 (12 h)
J
K
L
P110
M
DMSO
celastrol
withaferin A
BSA
palmitate
N
O
P
PA
celastrol
WFA
Q
water
893
DMSO
FCCP
R
S
T
FCCP
893

Figure EV2.

Figure EV3. Strategy for morphometric analysis of mitochondrial networks in vivo.

A Mitochondrial networks in freshly resected livers from mice fed a SD (left panels) or a HFD (right panels) imaged by NAD(P)H autofluorescence. Images are maximum intensity Z-projections derived from 6 Z-slices. Binarized mitochondrial networks were segmented to tag individual objects. Aspect ratio (tubule width/length) and roundness ((4 × area)/(π × width)) were measured on a per field basis. In violin plots (left), the center line is the median and the quartiles define the 25th to 75th percentile; data from the representative field of view shown. The scatterplots show the mean ± SD from 8 to 12 fields of view taken from each of 4 mice per group. The same strategy was applied to quantify hypothalamic and cortical mitochondria visualized with a citrate synthase antibody except that 6 fields of view were evaluated from each of 3 mice per group. Scale bar, 20 μm.

B NAD(P)H and MitoTracker Green (200 nM) co-localization in MEFs. Scale bar, 20 μm.

 © 2021 The Authors

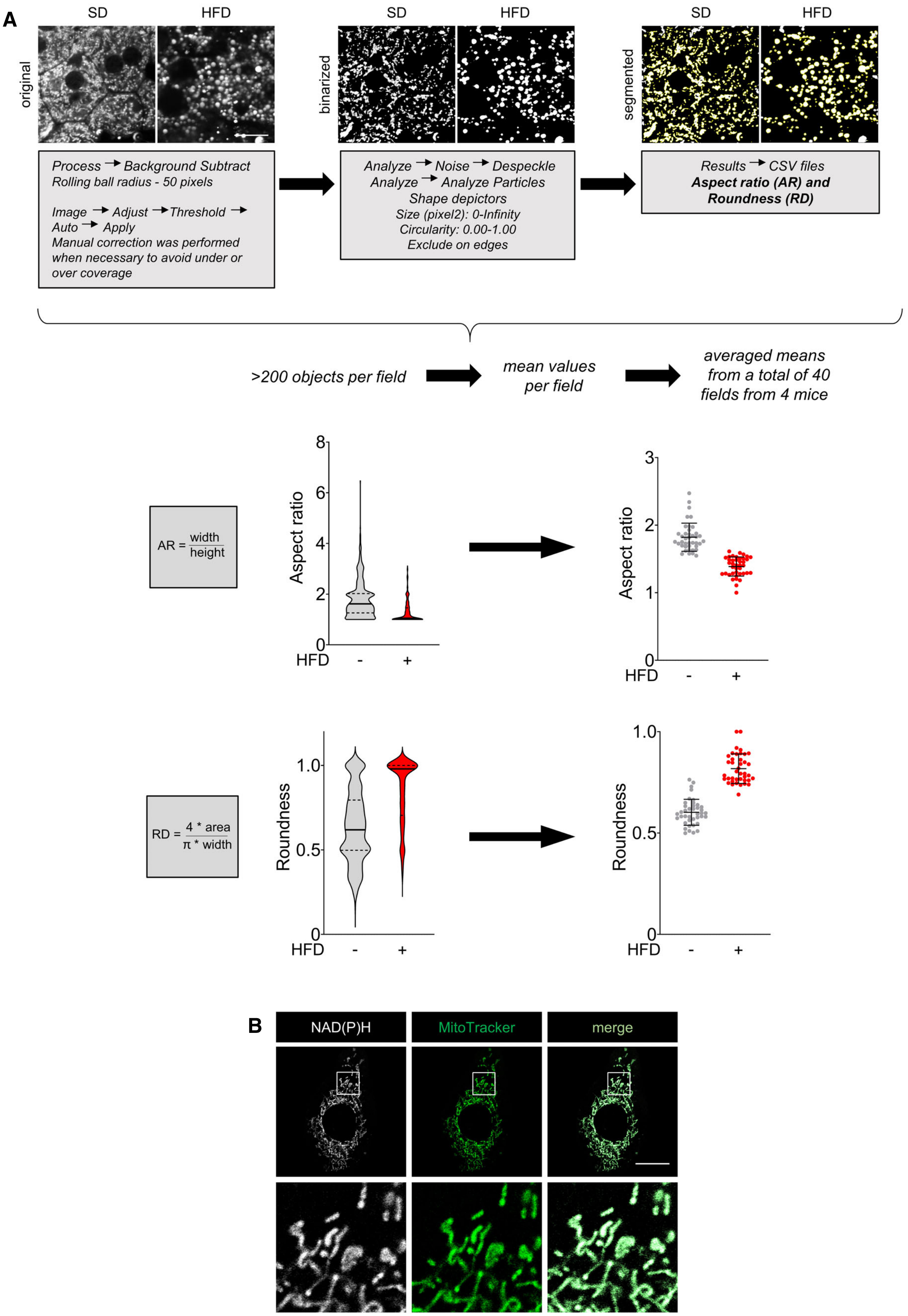
A
SD
HFD
original
binarized
segmented
Process → Background Subtract
Rolling ball radius - 50 pixels
Image → Adjust → Threshold → Auto → Apply
Manual correction was performed when necessary to avoid under or over coverage
Analyze → Noise → Despeckle
Analyze → Analyze Particles
Shape depictors
Size (pixel2): 0-Infinity
Circularity: 0.00-1.00
Exclude on edges
Results → CSV files
Aspect ratio (AR) and Roundness (RD)
>200 objects per field
mean values per field
averaged means from a total of 40 fields from 4 mice
AR = width / height
Aspect ratio
HFD
RD = 4 * area / π * width
Roundness
B
NAD(P)H
MitoTracker
merge

Figure EV3.

Figure EV4. SH-BC-893 reduces food intake and triggers weight loss without toxicity.

A, B Average energy expenditure using the Weir formula (A) or activity (B) from ZT12-ZT24 in the mice from Fig 6A–D that were fed a HFD for 10 weeks and then treated with either vehicle ($n = 8$) or 120 mg/kg 893 ($n = 6$–8) by gavage on days 1 and 3.

C–E Average daily distance run each day (C) or time spent on the wheel each day (D) or average daily time on wheels per mouse over the entire treatment period (E) in HFD-fed mice gavaged with vehicle ($n = 8$) or 120 mg/kg 893 ($n = 6$) Mon/Wed/Fri for 4 weeks.

F–I Relative (F,H) or absolute (G,I) fat (F,G) or lean (H,I) mass as measured by EchoMRI after 25 days of treatment in the mice shown in (C-E) and Fig 6L–N; $n = 6$–10.

J, K Daily food intake for the mice in Fig 6O–R. Mice were fed a chow diet or the HFD for 9-10 weeks then gavaged with vehicle or 120 mg/kg SH-BC-893 Mon/Wed/Fri for 3 weeks; ($n = 9$). Average food intake per mouse in the 24 h after each of 8 different treatments (indicated with arrows) is shown; mice were euthanized 4 h after the ninth treatment. On day 6 in (J), the University Lab Animal Resources technician unexpectedly added food to the hopper and intake could not be accurately quantified (break in line).

Data information: In all panels, mean ± SEM shown. Using unpaired t-tests (A, B, and E) or a one-way ANOVA with Tukey's correction (F-I), $*P \leq 0.05$; $**P \leq 0.01$; $***P \leq 0.001$. In F and H, comparisons are made to HFD + vehicle (*), HFD + 120 mg/kg SH-BC-893 (#), or HFD + vehicle + wheel ($).

© 2021 The Authors

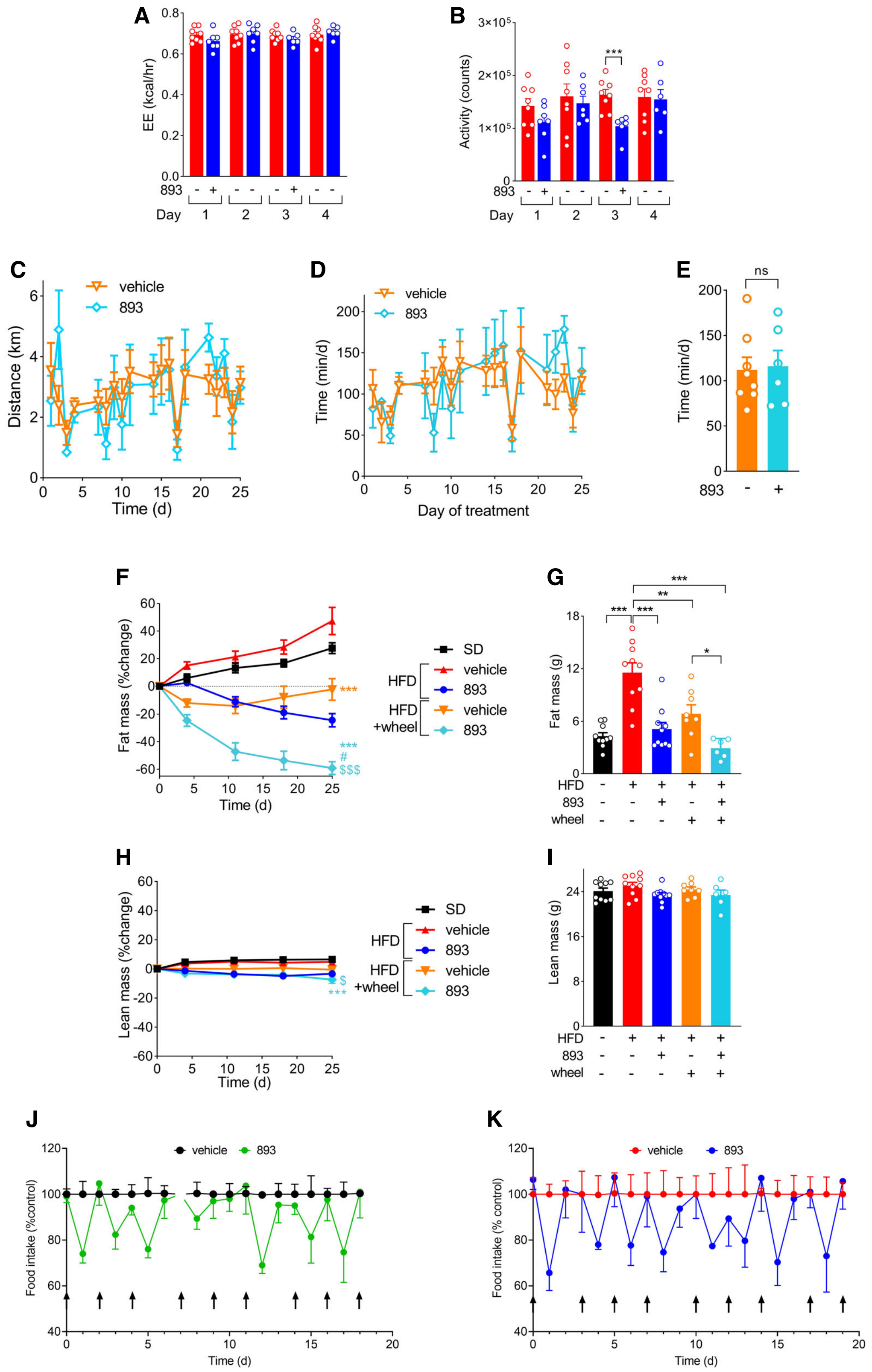
A
EE (kcal/hr)
893
Day
B
Activity (counts)
C
vehicle
893
Distance (km)
Time (d)
D
Time (min/d)
Day of treatment
E
ns
Time (min/d)
F
Fat mass (%change)
SD
HFD
vehicle
893
HFD +wheel
G
Fat mass (g)
HFD
893
wheel
H
Lean mass (%change)
I
Lean mass (g)
J
Food intake (%control)
K
Food intake (% control)

Figure EV4.

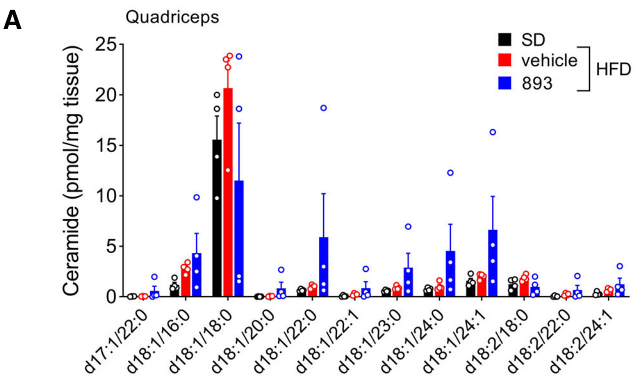

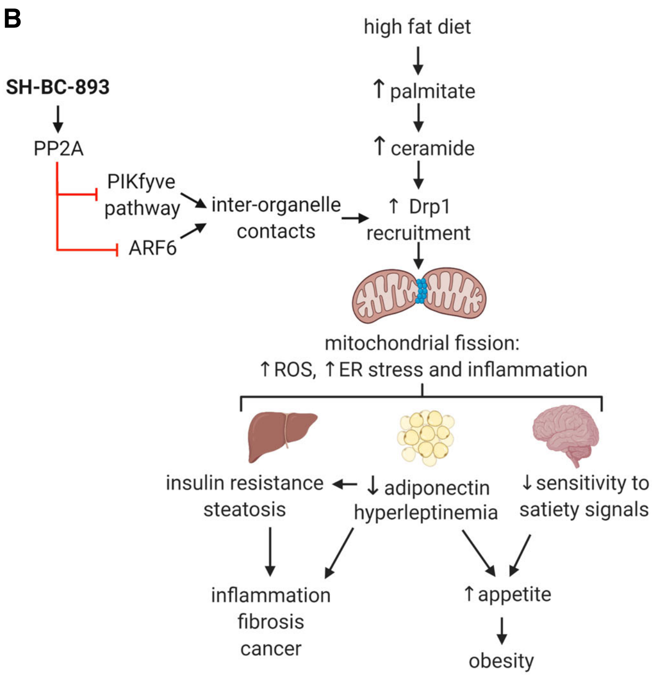

Figure EV5. SH-BC-893 effect on muscle ceramide and model figure.

A Ceramide levels in quadriceps muscle from the mice in Fig 7B–D at sacrifice; $n = 4$. Mean ± SEM. Using a one-way ANOVA with Tukey's correction for all ceramide species except d18:1/18:0 for which Brown–Forsythe and Welch ANOVA tests with Dunnett's correction was used due to unequal variance, $P > 0.05$.

B Model for anti-obesogenic effects of SH-BC-893.

 © 2021 The Authors
